# Fast Silencing Reveals a Lost Role for Reciprocal Inhibition in Locomotion

**DOI:** 10.1016/j.neuron.2012.10.040

**Published:** 2013-01-09

**Authors:** Peter R. Moult, Glen A. Cottrell, Wen-Chang Li

**Affiliations:** 1 School of Biology, University of St. Andrews, Bute Building, St. Andrews KY16 9TS, UK

## Abstract

Alternating contractions of antagonistic muscle groups during locomotion are generated by spinal “half-center” networks coupled in antiphase by reciprocal inhibition. It is widely thought that reciprocal inhibition only coordinates the activity of these muscles. We have devised two methods to rapidly and selectively silence neurons on just one side of *Xenopus* tadpole spinal cord and hindbrain, which generate swimming rhythms. Silencing activity on one side led to rapid cessation of activity on the other side. Analyses reveal that this resulted from the depression of reciprocal inhibition connecting the two sides. Although critical neurons in intact tadpoles are capable of pacemaker firing individually, an effect that could support motor rhythms without inhibition, the swimming network itself requires ∼23 min to regain rhythmic activity after blocking inhibition pharmacologically, implying some homeostatic changes. We conclude therefore that reciprocal inhibition is critical for the generation of normal locomotor rhythm.

## Highlights

► Silencing activity on one side stops fictive swimming ► Silencing activity on one side depresses reciprocal inhibition ► Motor rhythms resume ∼23 min after pharmacological block of inhibition ► Reciprocal inhibition is essential for maintaining swimming

## Teaser

It is widely thought that reciprocal inhibition merely coordinates the activity of antagonistic muscles. Using new methods which can silence nerve cells in milliseconds, Moult et al. show that reciprocal inhibition is also essential for generating locomotor rhythms.

## Introduction

Reciprocal inhibition is present in various neural circuits (Shepherd and Grillner, 2010) and has a well-established role in the coordination of antagonistic muscle activities. A century ago, Graham Brown proposed a half-center hypothesis to explain how spinal networks controlled stepping in decerebrate cats. In his proposal, reciprocal inhibition played a critical role in the generation of stepping rhythms as well as coordinating the activity of the two half-centers (Brown, 1911, 1914). The concept of half-centers initially referred to flexor and extensor spinal circuits but was then extended to refer to any antagonistic circuits including left and right sides of the spinal cord. Brown’s hypothesis has provided a basic framework for researchers to study neural rhythms that underlie various movements (Jankowska et al., 1967; Lundberg, 1981; Stuart and Hultborn, 2008; Katz et al., 2004; Arshavsky et al., 1993; Kristan et al., 2005; Grillner and Jessell, 2009; Ramirez et al., 2004). Although most circuits contain the basic anatomical half-centers, there has been little support for the requirement of reciprocal inhibition in locomotor rhythm generation as Brown originally proposed. Surgically dividing the two sides of the spinal cord in tadpoles (Kahn and Roberts, 1982; Li et al., 2010; Soffe, 1989), lamprey (Cangiano and Grillner, 2003, 2005; Cangiano et al., 2012; Hoffman and Parker, 2010), salamander (Ryczko et al., 2010), turtle (Samara and Currie, 2008; Stein et al., 1998), mouse (Hinckley et al., 2005; Kwan et al., 2009), and rat (Ozaki et al., 1996) failed to abolish unilateral bursting. On the other hand, motor bursts remained in intact preparations when both reciprocal and ipsilateral inhibition were blocked by strychnine (Cangiano and Grillner, 2003; Cohen and Harris-Warrick, 1984; Guertin and Hounsgaard, 1998; Li et al., 2010; Rioult-Pedotti, 1997; Soffe, 1989; Bracci et al., 1996; Cowley and Schmidt, 1995; Droge and Tao, 1993; Hinckley et al., 2005; Kremer and Lev-Tov, 1997; Ozaki et al., 1996). In most cases, the motor bursts in the absence of both reciprocal and ipsilateral inhibition differed from the rhythms in intact cords in terms of frequency and regularity, especially in rodents. Although it is tempting to draw a general conclusion that reciprocal inhibition is not needed in the generation of basic locomotor rhythms from these studies, the possibility for compensatory changes (for reviews, see Davis and Bezprozvanny, 2001; Marder and Goaillard, 2006) that may cause rhythmicity cannot be excluded (Hoffman and Parker, 2010).

*Xenopus* tadpole swimming is controlled by neural circuits in the spinal cord and caudal hindbrain, which are symmetrical on the left and right sides connected by reciprocal inhibitory commissural interneurons (cINs) (Li, 2011; Roberts et al., 2010). We have devised two methods capable of depressing reciprocal inhibition on millisecond scales in this study. We found that the two sides of the tadpole swimming circuit relied on each other during swimming, supporting a critical role for reciprocal inhibition in the generation of locomotor rhythm.

## Results

### Yellow Light Stopped Swimming in Tadpoles Expressing ArCh on One Side

**Figure 1:**
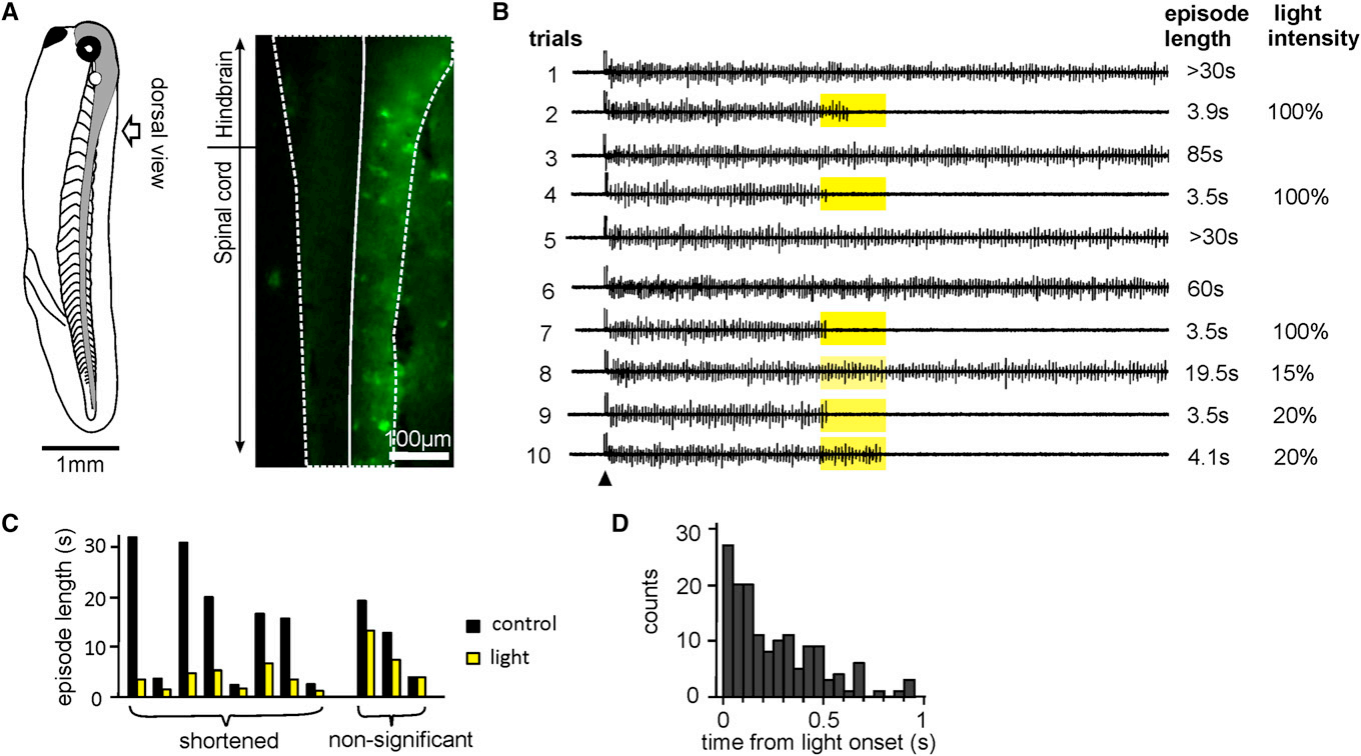
Activation of ArCh in Neurons on One Side of the Tadpole Stops Swimming (A) Left: diagram of a stage 37/38 tadpole viewed from the side; the CNS is shown in gray. Right: ArCh-GFP expression in a tadpole at the same stage after injecting ArCh cRNAs into a blastomere at the two-cell stage. The preparation is viewed from above, after removing the skin and muscle; the right (GFP+) and left sides of the CNS are delineated. (B) Ten consecutive trials showing the effect of 1 s periods of illumination on swimming episode length for the tadpole in (A) (recordings are from the left m.n.). Arrowhead points at time of skin stimulation. One hundred percent light intensity is 10 mW/mm^2^. (C) Average episode lengths with illumination in eight out of 11 tadpoles (paired columns) were significantly shortened. The first pair of columns on the left is a summary of data in (B). (D) Distribution of the time taken for a 1 s period of illumination to stop swimming in 149 successful trials.

First, we injected green fluorescent protein (GFP)-tagged Archaerhodopsin-3 (Chow et al., 2010) (ArCh, a light-driven outward proton pump from *Halorubrum sodomense*) complementary RNA (cRNA) into one blastomere in the two- to eight-cell stage embryos. Blastomere lineage fate is uniquely predetermined in *Xenopus laevis* from the one-cell stage (Moody, 1999). So injection into one blastomere leads to specific ArCh-GFP expression in neurons of only one side of the nervous system (Figure 1A). Expression could be seen clearly in many somata but did not allow anatomical identification of different types of neurons. Activation of ArCh using yellow light (peak wavelength: 585 nm) quickly hyperpolarizes neurons (Chow et al., 2010) (time constant for inhibition at rest is 65.7 ± 14 ms, n = 7, Figure 4A). We chose tadpoles in which ArCh was expressed in the right side of the nervous system, observed by the tagged GFP, for testing the effect of yellow light on swimming episode lengths. This allowed recording of motor nerve (m.n.) discharges from the (ArCh-GFP-negative) left side. Yellow light was applied 1–5.5 s after swimming was initiated. Illumination trials were alternated with control episodes so that we could conveniently compare them using either paired t tests or Wilcoxon signed-rank tests, depending on the distribution of measurements of individual recordings. Yellow light shortened swimming episodes significantly in 8 out of 11 tadpoles monitored from m.n. recordings on the left (n ≥ 5 trials, p < 0.05 in each of the eight tadpoles, Figures 1B and 1C). Trials with illumination (0.9–6.2 s, depending on time of illumination) were 38.3% ± 5.9% of their immediate control episode length (1.5–85 s, p < 0.001, related sample Wilcoxon signed-rank test, n = 67 trials). Critically, swimming stopped during the illumination period, with a short delay from the onset of illumination to the last m.n. burst (median 0.19 s, range 0–0.94 s; or a median of two swimming cycles ranging from 0 to 17, 149 trials analyzed, Figure 1D).

### Hyperpolarizing Single dINs Stopped Swimming

**Figure 2:**
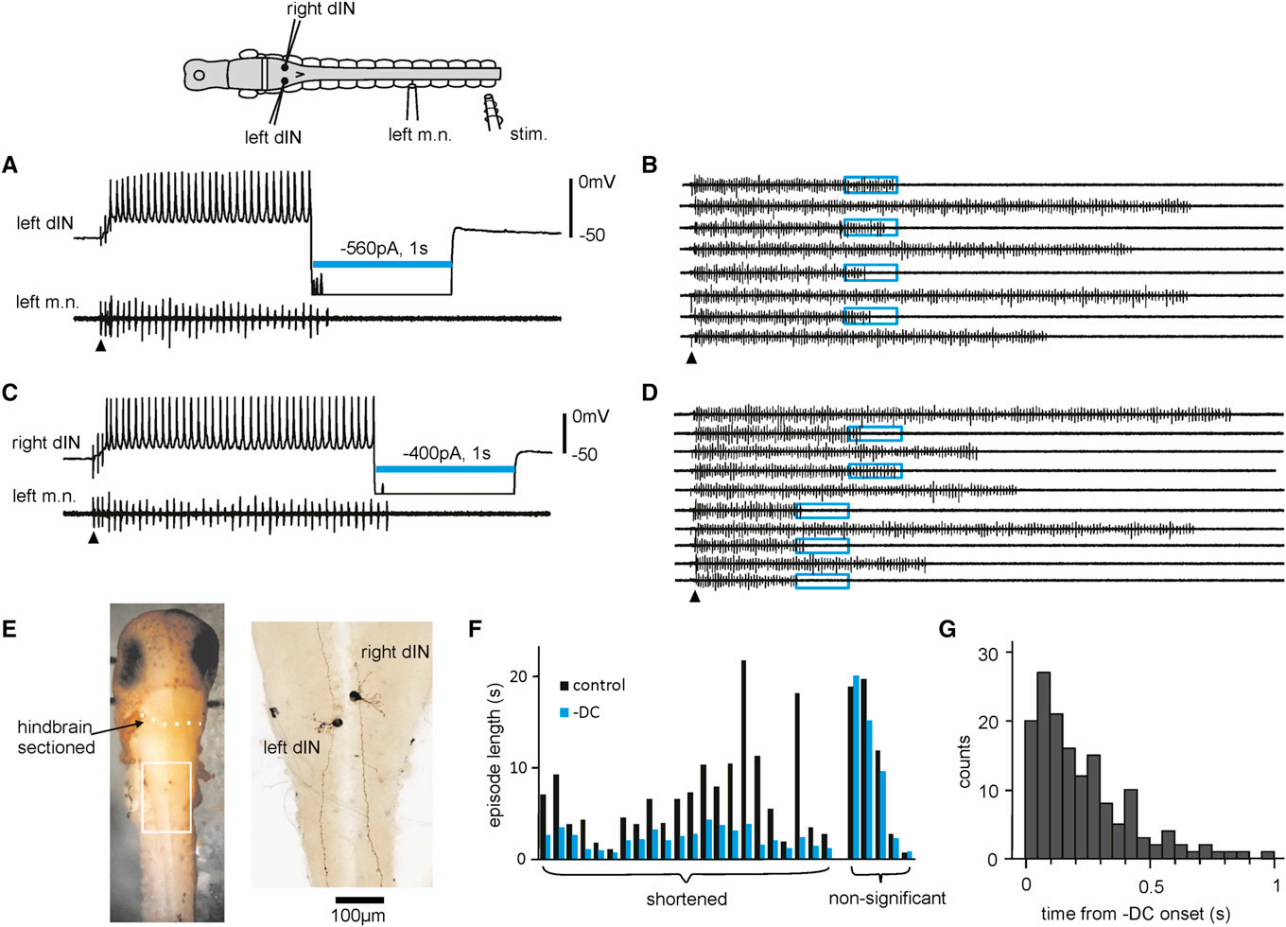
Swimming Stopped Abruptly when Large Hyperpolarizing Currents Are Injected into Single dINs (A) Injecting −560 pA into a dIN on the left side stopped swimming. (B) Repetitive trials of 1 s −DC injection (blue boxes) into the dIN shown in (A) alternated with controls. (C) Injecting −400 pA into a dIN on the right side also stopped swimming. (D) Repeated 1 s −DC injections (blue boxes), as shown in (C), were alternated with controls. dINs in (A) and (C) are recorded simultaneously, but only one recording trace is shown to simplify illustration. (E) Neurobiotin staining of the dINs recorded in (A) and (C). Left: dorsal view showing the location of dINs in the caudal hindbrain (dotted line marks location of cross-section). Right: the anatomy of the two dINs with their ipsilateral axons magnified from the boxed area in the left photo. Arrowhead points at the time of skin stimulation in (A)–(D). Recordings in (A) and (C) are off scale during −DC. (F) Average episode lengths are shortened by −DC injections in 22 out of 27 dINs (cf. controls). (G) Distribution of the time taken for a 1 s −DC to stop swimming in 152 successful trials. Top diagram is a dorsal view of the CNS with muscles and electrodes. Hindbrain was sectioned at the white line. m.n., motor nerve recording; Stim., stimulating electrode.

The tadpole swimming circuit contains just one type of excitatory premotor interneurons (descending interneurons [dINs]) (Li, 2011; Roberts et al., 2010). dINs possess only ipsilateral axons and fire the earliest on each swimming cycle. Their activity drives the firing of other types of neurons (Soffe et al., 2009). We recently showed that dINs are extensively electrically coupled to each other (Li et al., 2009). Injecting large hyperpolarizing currents (−DC) into a single dIN instantly lowers swimming frequency and sometimes stops swimming (Li and Moult, 2012). There are about 200 dINs on each side of the spinal cord and hindbrain. The −DC may spread into a subset of dINs in the hindbrain, stop their firing, and affect swimming. We injected −DC larger than previously used (−0.4 to −1 nA, 1 s) into single dINs in order to shut off the excitatory drive to the swimming circuit and stop the activity reliably on the side where the dIN was recorded. As in the light illumination experiments above, we alternated episodes with −DC injections with controls to assess the effects of –DC injections in each tadpole. Swimming activity was monitored by recording m.n. discharges or another neuron on the opposite side. In 22 out of 27 dINs recorded in the caudal hindbrain area, injecting −DC 0.5–4.5 s after the beginning of swimming reliably stopped swimming (n ≥ 5 trials and p < 0.05 in each dIN, paired t test or Wilcoxon signed-rank test applied to individual recordings, Figures 2A, 2C, and 2E). Swimming episodes were shortened (0.5–5.2 s, median 2.1 s) by −DC injections into dINs to 44.5% ± 3% of their immediate controls (0.9–30 s, median 4.4 s, n = 148 trials, p < 0.001, related sample Wilcoxon signed-rank test, Figures 2B, 2D, and 2F). Similarly to the light-silencing experiments, swimming stopped rapidly after −DC injections (median time from −DC onset to the last m.n. burst was 0.18 s, range 0–0.97 s; median number of swimming cycles was 2, range 0–13, 152 trials analyzed, Figure 2G).

### Neuronal Firing during Swimming Was Depressed by One-Sided Silencing

**Figure 3:**
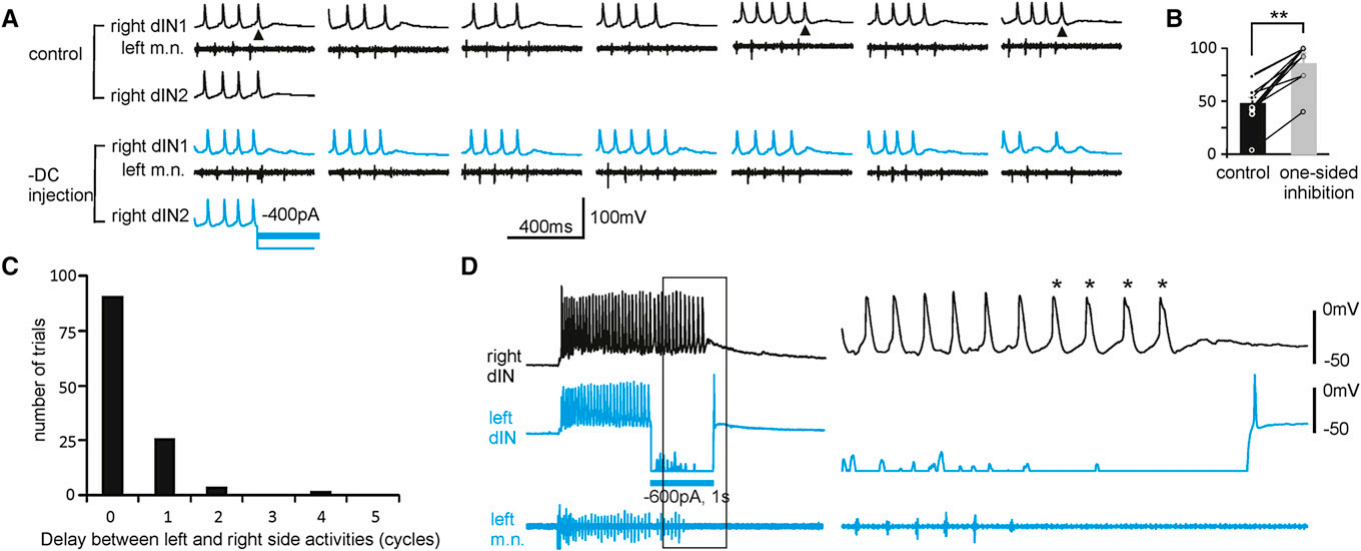
Activity Normally Stops First on the Silenced Side (A) The activity on both sides in the last few cycles in the control and when the right side dINs were injected with −DC (seven trials each, dIN2 activity only shown for the first trial). Arrowheads in the control point to examples where the left side activity stops first. (B) Percentages of control swimming episodes with activity ending first on the left (black) and of trials with activity stopping first on the inhibited side in one-sided silencing (gray). ^∗^p < 0.05, ^∗∗^p < 0.01. (C) The distribution of delay between left and right side activity in 123 trials in which activity stopped first on the suppressed side. A half-cycle delay is represented by “0.” (D) One of the two trials in which dIN activity on the opposite side carried on for four more “cycles” (^∗^, cf. C) after the activity on the suppressed left side has stopped. Recordings on the right are expanded from the boxed area. Recording of the left dIN during −DC was off scale.

The one-sided silencing experiments (light illumination or −DC injection) therefore show that swimming rhythms on one side are critically dependent on the activity in the other. We next investigated mechanisms that could enable one-sided silencing to stop swimming. We asked whether the activity stopped on a particular side first. The neuronal activity stopped first on the suppressed side in most cases (88.7% ± 5.7%, 67 light illumination trials in three tadpoles plus 71 −DC trials in eight dINs, Figures 3A and 3B). This normally took place within less than one cycle after the last m.n. burst on the suppressed side (Figure 3C), though occasionally extra firing was observed (Figure 3D). In contrast, there was no preference in control tadpoles in which swimming activity stopped spontaneously (48.5% ± 7% of 177 episodes with left side activity stopping first in eight tadpoles, p < 0.01, related sample Wilcoxon signed-rank test, Figure 3B).

**Figure 4:**
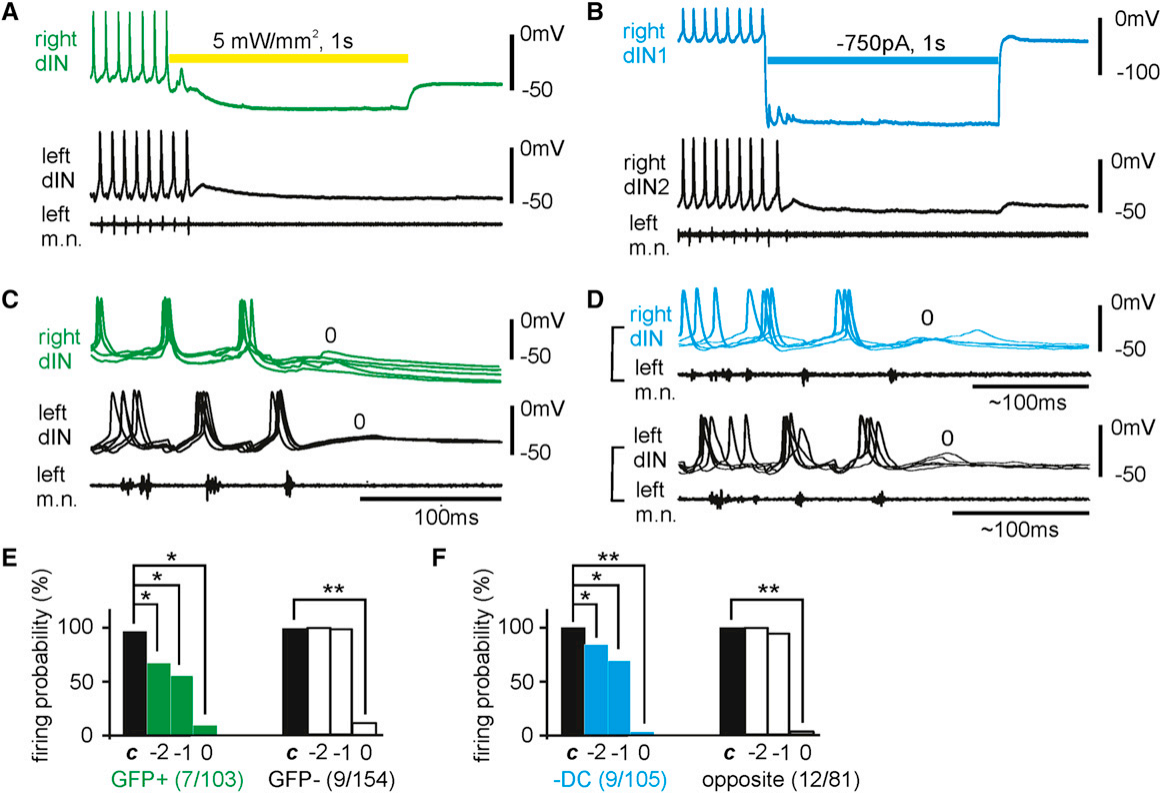
The Firing Probability of Neurons before and during One-Sided Silencing (A) Simultaneous recordings from a dIN on each side of the cord and also from a left m.n. to show the effect of illumination (yellow bar). (B) Simultaneous recordings from two other dINs and left m.n. with −DC injection into dIN1 (blue bar). (C) Five superimposed examples showing the firing of two dINs at the end of episodes in which light stopped swimming within three cycles. (D) Five superimposed recordings of a dIN from the side with −DC injection (blue, top traces) and of another dIN from the opposite side in a separate recording (bottom traces). The recording of the dIN injected with −DC is not shown. In (C) and (D), traces are aligned to the last m.n. bursts and some traces are rescaled horizontally for clarity. Cycle 0 is the period after the last m.n. burst. In (A–D), green traces are recordings from the GFP+ side; blue traces are recordings from the side with −DC injections. (E and F) Summary of the average firing probability in the last three cycles and controls (***c*** is the average of five cycles before silencing). Numerals in brackets are number of cells/trials. We define firing probability of an individual neuron as the percentage of swimming cycles with neuronal firing. See also Figure S1.

Rhythmically firing neurons typically fired action potentials reliably in a one spike per cycle manner during swimming, giving a near 100% firing probability in controls. During one-sided silencing, the firing probability decreased. We compared neuronal firing probability in the last three cycles at the end of each swimming episode with controls (n = 37, of which 31 were dINs). We defined “cycle 0” as the period (∼100 ms) immediately after the last m.n. burst. Cycles −1 and −2 were the last and second last cycles, respectively. One-sided silencing reduced firing probability in neurons recorded from the suppressed side in all three cycles. In cycle 0, it was 6.9% for light illumination (range: 0%–25%, 7 cells/103 trials) and 0% for −DC injections (range: 0%–6.3%, 9 cells/105 trials). In cycles −1 and −2, they were 56.6% ± 11.2% and 68.3% ± 11.3% for light illumination and 70% ± 8.5% and 85.5% ± 5.4% for −DC injections, respectively. In the opposite side, firing probability only dropped in cycle 0. It was 0% for light silencing (range: 0%–40%, 9 cells/154 trials) and 0% for −DC injections (range: 0%–50%, 12 cells/81 trials, p < 0.05, Wilcoxon signed-rank test in each case, Figures 4A–4F). Neuronal firing probability in animals in which one-sided silencing failed to stop swimming did not drop significantly (see Figure S1 available online). The significant drop in firing probability in neurons on the suppressed side in cycle 0 means that the opposite side would receive much smaller reciprocal inhibition, which might have resulted in reduced firing probability there too.

### One-Sided Silencing Stopped Swimming by Depressing Reciprocal Inhibition

**Figure 5:**
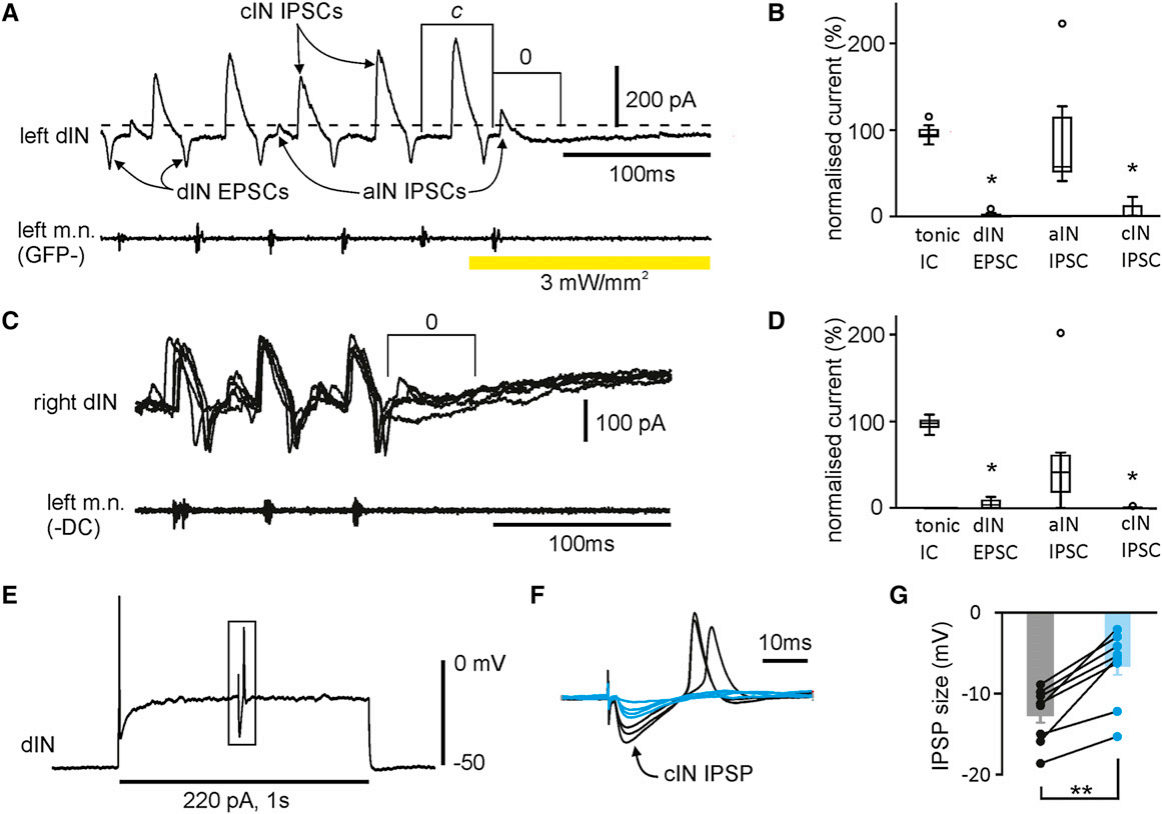
One-Sided Silencing Depressed cIN Inhibition and dIN Excitation and Necessity for cIN Inhibition in dIN Rebound Firing (A) The last cycles of a swimming episode, in which light (yellow bar) stopped swimming within one cycle. Different synaptic currents are labeled (*c* is used as a control cycle). (B) Normalized synaptic currents in dINs in cycle 0, as shown in (A), in light silencing trials (eight dINs, 53 trials). Tonic inward current (IC) was measured as the difference between the clamping current at rest (dashed line in A) and the current level just before each cIN IPSC. (C) Five superimposed trials with −DC injections, aligned to the last m.n. burst, showing synaptic currents in cycle 0. (D) Normalized synaptic currents in dINs in cycle 0 in −DC injection experiments (seven dINs, 51 trials). Synaptic currents are normalized to those in control cycles in (B) and (D). All recordings are from the ArCh-GFP negative side or the side without −DC injections into dINs. (E) dIN usually fires a single spike at the onset of a depolarizing pulse (220 pA, 1 s) but can also fire on rebound. (F) The boxed area is expanded to show rebound spikes following cIN IPSPs (seven trials overlayed). IPSPs failing to evoke dIN rebound spikes are blue. (G) The size of cIN IPSPs that evoked dIN rebound firing (black and gray) and the size of IPSPs that failed to evoke firing (blue). Error bars represent SE. ^∗∗^p < 0.01.

We then analyzed the synaptic currents in dINs in cycle 0 on the opposite side to identify the cause of the failure of dIN action potentials. Rhythmic neurons in the tadpole swimming circuit receive three types of rhythmic synaptic inputs (dIN excitatory postsynaptic current [EPSC], ascending interneurons [aINs] inhibitory postsynaptic current [IPSC], and cIN IPSC) and tonic inward currents (Li and Moult, 2012). We clamped the membrane potential of dINs at ∼−20 mV, so that these currents could be monitored simultaneously. Rhythmic synaptic currents were separated based on their different timing in the cycle: on-cycle dIN EPSC immediately before ipsilateral m.n. bursts, midcycle cIN IPSC onset about the middle between two adjacent dIN EPSCs, and early-cycle aIN IPSCs between dIN EPSCs and cIN IPSCs (Figure 5A; Li et al., 2010). Trials in which one-sided silencing stopped swimming within three cycles were chosen for analyses to enable comparisons of the currents with controls before silencing. Synaptic currents during silencing periods were normalized to control levels in individual recordings and averaged between neurons (light silencing: n = 8 cells, 53 trials, −DC: 7 cells, 51 trials). In cycle 0, cIN IPSCs (light silencing: median 0, range 0%–22%; −DC injections: median 0, range 0%–1%) and dIN EPSCs (light silencing: median 0, range 0%–8.3%; −DC: 4.7% ± 2%) dropped to near 0% of their controls (p < 0.05, Wilcoxon signed-rank test in each case). In contrast, tonic inward currents (light silencing: 96.9% ± 3.9%; −DC: 96.8% ± 2.8%) and aIN IPSCs (light silencing: 93.4% ± 24.6%; −DC: median 37, range 0%–202%) did not change significantly (p > 0.05, Wilcoxon signed-rank tests in both cases, Figures 5A–5D). aIN activity is driven by the activity of ipsilateral dINs in the preceding cycle. The lack of change in aIN IPSCs in cycle 0 is consistent with the observation above that neuronal activity on the nonsilenced side is not suppressed in cycles −1 and −2 (Figures 4G and 4H).

### dIN Rebound Firing Requires Sufficient Reciprocal Inhibition

Two mechanisms can support regenerative dIN firing during swimming: rebound firing after inhibition from cINs and n-methyl-d-aspartate (NMDA) receptor-dependent pacemaker firing, if inhibition is pharmacologically blocked (Li, 2011; Li et al., 2010; Soffe et al., 2009). Our results above have revealed that one-sided silencing suppressed cIN IPSCs on the opposite side. This led to the failure of dIN rebound firing and the consequent disappearance of dIN EPSCs, which drive neuronal activity. We tested the relationship between cIN inhibition strength and the probability of dIN rebound firing by stimulating cINs in the opposite side of the spinal cord directly, with excitatory neurotransmission blocked (see Experimental Procedures). Background depolarization was maintained by 0.5–1 s superthreshold DC injections into dINs. cIN inhibitory postsynaptic potential (IPSP) amplitude was altered by adjusting the stimulating current intensity. IPSPs, which failed to evoke dIN rebound firing (−6.6 ± 1.5 mV), were 49.2% ± 8.1% of those that did evoke dIN rebound firing (−12.6 ± 1.1 mV, n = 8 dINs, p < 0.001, paired t test, Figures 5E–5G), confirming that reciprocal inhibition needs to be sufficiently large to evoke dIN rebound firing and thus sustain activity.

### dIN Pacemaker Properties in Intact Tadpoles

**Figure 6:**
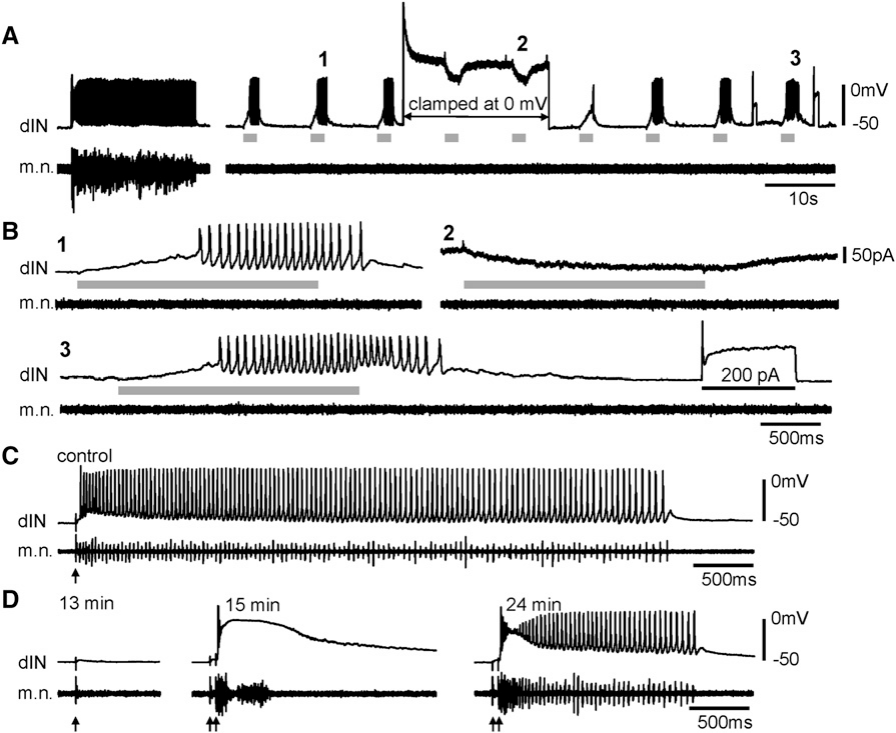
dIN Pacemaker Firing in an Intact Tadpole and Recovery of Motor Rhythms after Inhibition Blockade (A) The activity of a dIN during swimming in an intact tadpole (left) and after short NMDA applications using microiontophoresis (1.3 nA for 2 s, gray bars). The short period of voltage-clamp recording is marked (arrowed line). (B) NMDA-application trials (1–3) in (A) at a faster time scale. The dIN only fires a single spike to DC injections either before (100 pA) or after (200 pA, black bar) trial 3. Note the absence of m.n. activity and fast synaptic currents in NMDA application trials. (C) The activity of a dIN in control swimming in a tadpole cross-sectioned at the fifth and sixth rhombomere levels. (D) dIN and m.n. activity at different time after bath-applying 2.5 μM strychnine and 20 μM SR95531. Recovery period for motor rhythms in this tadpole is 12 min. Arrows indicate time of skin stimulation (artifacts reduced for clarity).

Occasional extra spikes on the uninhibited side after one-sided silencing suggest that pacemaker firing capability is present in at least some dINs. We next applied NMDA locally in intact tadpoles to see if most dINs could fire like pacemakers. Microiontophoresis via high-resistance microelectrodes next to the recorded neuron was employed to restrict its localization (Li et al., 2010). Tests were carried out after moving the microelectrode around slightly to find the most sensitive spot so that iontophoresis currents could be minimized (−DC ≤ 2 nA). The results showed that all dINs could fire repetitively to short 2 s applications of NMDA (n = 183 trials in 10 dINs), though they typically fire a single spike to current injections at rest (Figure 6A). This type of firing is most likely pacemaker firing because, during and shortly after the application, there was no m.n. activity (all 183 trials, except for one tadpole, for which swimming occurred in three trials) or evoked synaptic currents when the recording was briefly switched to voltage-clamp mode (∼0 mV, 38 trials in six tadpoles, Figure 6B, four trials in two tadpoles with some unpatterned IPSCs).

**Figure 7:**
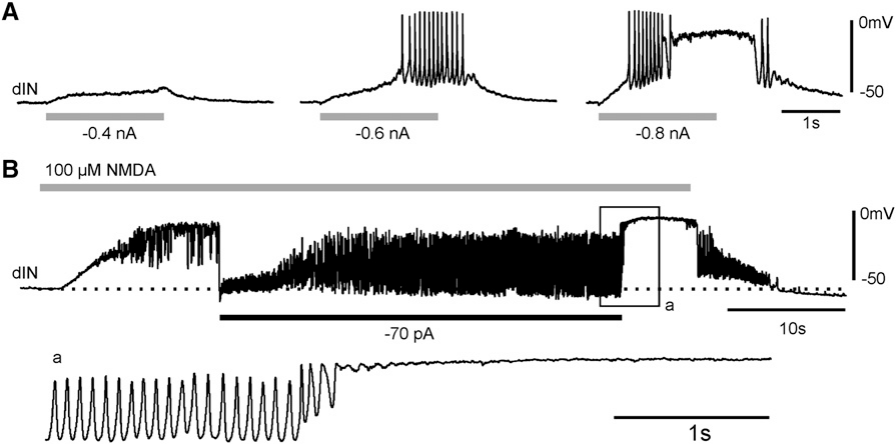
Hyperexcitation Blocks dIN Pacemaker Properties (A) The responses of a dIN to three consecutive applications of NMDA at different microiontophoresis currents (gray bars). The right hand trial results in repetitive firing followed by seizure-like depolarization. (B) A dIN’s response to microperfusion of 100 μM NMDA in TTX (gray bar). Injecting hyperpolarizing current (−70 pA) into the dIN reveals reliable oscillations, which quickly change to seizure-like depolarization at the current withdrawal. The boxed area (a) is expanded below. The dotted line indicates the resting membrane potential level.

In accord with this, NMDA-induced tetrodotoxin (TTX)-resistant 10 Hz oscillations, which underlie repetitive dIN pacemaker firing, could be recorded in intact tadpoles as soon as 100 μM NMDA was microperfused (44–300 s after 0.4 μM TTX blocked action potentials; n = 14 dINs; for oscillation examples, see Figure 7B).

### Time Course for the Emergence of Pacemaker-Driven Rhythms

The above results show that pacemaker firing properties are normally present in dINs. Our previous study showed that pacemaker properties in dINs (Li et al., 2010) could sustain swimming-like rhythms after surgical separation of the two sides of the spinal cord and pharmacological blockade of inhibition. Pacemaker firing, however, failed to support motor rhythms in the fast silencing experiments. This implies that, in normal swimming, this type of pacemaker firing does not play a dominant role. After inhibition/rebound firing is blocked, it takes time for pacemaker properties to emerge as the driving force for motor rhythm. We attempted to reveal the time course for this by blocking inhibition using 2.5 μM strychnine and 20 μM SR95531 (gabazine), because the surgery results in at least a 20 min gap before recordings. Tadpole tail skin was stimulated every 30 s to monitor m.n. outputs continually. After antagonist application, the amplitude of cIN IPSCs in the recorded neurons, monitored by simultaneous voltage-clamp recordings, fell to indiscernible levels within 2 min (n = 8 neurons). In one out of eight tadpoles, swimming could only be started by double-pulse skin stimulation in controls. Rhythmic motor bursts could be evoked without a clear break throughout antagonist application (31 min), although the episodes were shortened (1.4 ± 0.1 s from 9.8 ± 3.2 s in control, p < 0.01, t test). In the other seven tadpoles, rhythms evoked by single-pulse skin stimulation disappeared after 2–7 min and did not return up to 43 min after drug application (265 trials, Figure 6D), except that in two trials, rhythmic activity was observed and, in three other trials, seizure-like neuronal depolarization at ∼−5 mV and tonic nonrhythmic m.n. bursts were induced at the early stage of block. We used repetitive skin stimulation (normally two pulses at 30 Hz) with the same intensity to test whether this could rescue motor rhythms after single-pulse stimulation had failed. Rhythmic motor bursts recovered after some time, but average episode lengths (1 s, range: 0.7–5.7 s) were shorter than control (11.7 ± 3 s, p < 0.05, Wilcoxon signed-rank test, Figures 6C and 6D). The recovery period, from the first three consecutive rhythm failures to the three consecutive trials with rhythms evoked by repetitive skin stimulation, was 23.1 ± 4.3 min (range: 5.5–37, n = 7). During the recovery period, the majority of responses to two-pulse skin stimulation were seizure-like depolarization to ∼−5 mV and tonic bursts in m.n. (77 out of 108 trials, Figure 6D). There was occasional rhythmic activity in six of the seven tadpoles (11 out of 108 trials). No obvious response was seen in the other 20 trials.

The failure of dINs to fire rhythmically during the recovery period may result from failed dIN pacemaker properties resulting from depolarization block, e.g., as seen in midbrain dopamine neurons in response to acute excitation (Tucker et al., 2012). In accord with this view, increasing NMDA iontophoresis currents can convert repetitive dIN firing to sustained depolarization at ∼−5 mV (40 trials in six dINs, Figure 7A). On the other hand, negative currents were often needed in dINs to hyperpolarize membrane potential from seizure-like depolarization to get reliable TTX-resistant oscillation (n = 21 trials in 12 dINs, Figure 7B).

## Discussion

**Figure 8:**
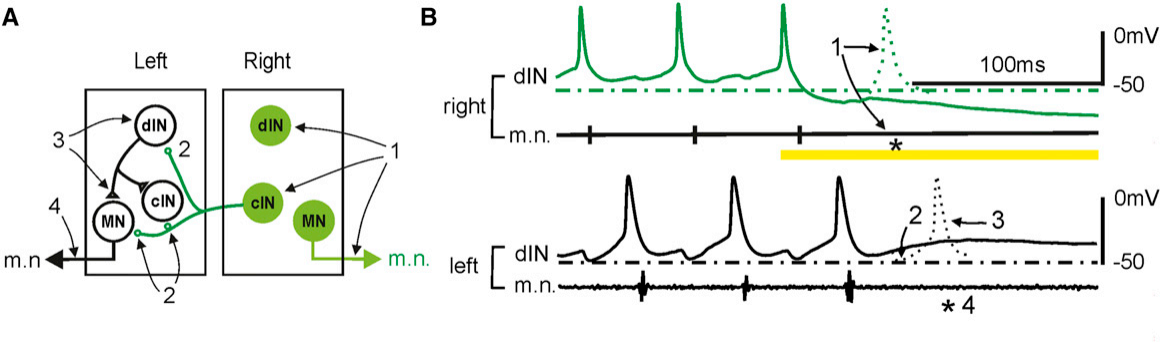
Failure in dIN Rebound Firing May Underlie the Cessation of Swimming after One-Sided Silencing (A) A simplified swimming circuit (circle inhibitory, triangle excitatory, synapses). (B) Simultaneous recordings from a right and a left dIN and also a left m.n. To explain the sequence of events after light silencing, the timing of right m.n. bursts is shown schematically. Dashed lines indicate resting membrane potential levels. Dotted traces in (B) of whole-cell recordings show predictions of the sequence of events (1–4, cf. A) if light illumination (yellow bar) had failed to inhibit the activity in cycle 0 on the GFP+ side (green symbols and traces). Asterisk indicates the timing of m.n. bursts had they occurred. See the main text for more details.

This study shows that the two sides of spinal cord and hindbrain depend on each other to maintain the normal swimming rhythm (10–25 Hz) via reciprocal inhibition. Light activation of ArCh or −DC injections into single dINs: (1) stops neuronal firing on the suppressed side, (2) weakens cIN inhibition from the suppressed side, (3) results in dIN rebound failures and (4) leads to the cessation of swimming on the opposite side (Figures 8A and 8B). cINs, which are rhythmically active during swimming, have been shown to be inhibitory in paired recordings (Dale, 1985; Li et al., 2007). Intracellular recordings from neurons below the hemisection also confirmed that the neurons just received rhythmic inhibition from the intact side (Soffe and Roberts, 1982). Some excitatory sensory interneurons also have commissural axons, but they are not active during swimming (Li et al., 2007; Roberts et al., 2010). There is no common command neuron driving neural activities on both sides. Instead, excitatory drive comes from dINs located on each side extending from the spinal cord to the caudal hindbrain (Li et al., 2004, 2006, 2010; Soffe et al., 2009). We show in this study that silencing the activity on one side also quickly stops activity on the contralateral side. The injections of −DC into dINs removed the excitatory drive in cINs, thus working indirectly to depress cIN activity. Light inhibition also directly depresses cIN activity. Both methods led to specific depression of cIN IPSCs and the subsequent disappearance of dIN EPSCs due to failure of rebound on the other side. This indicates that reciprocal inhibition plays a critical role in the generation of the normal swimming rhythm, as suggested in tadpole swimming models (Roberts and Tunstall, 1990; Sautois et al., 2007). This matches tadpole swimming behavior, in which the two sides always stop contracting within one swimming cycle. However, it contradicts previous observations (Soffe, 1989; Li et al., 2010) that swimming-like rhythms can be generated in hemicord preparations.

### Homeostatic Plasticity and the Role of Reciprocal Inhibition in Lamprey Swimming

Redundant mechanisms or homeostatic plasticity have been found in many systems (Marder and Goaillard, 2006; Davis and Bezprozvanny, 2001; Desai et al., 2002; Echegoyen et al., 2007; Turrigiano, 2007; Sakurai and Katz, 2009; Hoffman and Parker, 2010; Rossignol et al., 2004), and they can be upregulated when normal neural activity is disrupted. Homeostatic plasticity develops over different time scales but can occur within 5–10 min (Frank et al., 2006). In most previous studies, strychnine application was used, or in cases of axial swimming networks midline cuts (hemicord) were made, to remove reciprocal inhibition. These methods take at least several minutes to work, during which homeostatic, compensatory mechanisms can conceivably occur (Frank et al., 2006).

Homeostatic changes can complicate the interpretation of experimental results, especially under different experimental conditions. For example, strychnine application was initially shown to cause tonic irregular m.n. activity (Grillner and Wallén, 1980). This was overlooked after a second study (Cohen and Harris-Warrick, 1984), in which synchronous activity on both sides of lamprey spinal cord was observed in the presence of strychnine, suggesting reciprocal inhibition is not needed in unilateral bursting. Laser ablation of commissural interneurons in intact spinal segments, which disrupts action potential propagation within minutes, later revealed that reciprocal inhibition was necessary in lamprey swimming rhythm generation (Buchanan and McPherson, 1995). More recent studies, however, found that hemisegments were capable of generating both fast (2–12 Hz) and slow (0.1–0.4 Hz) motor rhythms (Cangiano and Grillner, 2003, 2005). A more detailed examination of neuronal properties revealed that excitability in ipsilateral excitatory interneurons and motoneurons was enhanced 30–60 min after hemisectioning lamprey spinal cord, which coincides with the time course for the development of NMDA-induced slow rhythms (Hoffman and Parker, 2010). This suggests that the slow rhythms in lamprey hemicord preparations may result from homeostatic changes in the network. The fast rhythms are about two to three times faster than fictive swimming in intact cords and they persisted in strychnine and so would be independent of both reciprocal and ipsilateral inhibition (Cangiano and Grillner, 2003). Although they can be induced as soon as recording is possible after hemisectioning (6–11 min) (Cangiano et al., 2012), similar examination of neuronal properties using intracellular recordings would not be practical within such a short time window. Since some homeostatic changes can take place within a few minutes (Frank et al., 2006), it remains undetermined whether homeostatic changes could contribute to the fast rhythms in lamprey hemispinal segments.

### Tadpoles Swimming Rhythm Generation Mechanisms

In the tadpole swimming circuit, rebound mechanisms were proposed for the maintenance of swimming based on analyses of synaptic events during swimming and the firing property of dINs at rest (Li et al., 2006; Soffe et al., 2009). When hemisections were made or strychnine was applied to remove reciprocal inhibition, however, rhythmic activity at slightly higher frequencies than that in swimming persisted (Soffe, 1989). This has led to the proposal of dIN pacemaker firing in supporting swimming rhythms (Li et al., 2010; Li, 2011). In this study, one-sided silencing specifically removes cIN inhibition but leads to failure of rhythmic activity on both sides. This suggests dIN rebound firing is the normal operating mechanisms for swimming, because only rebound mechanisms rely on cIN inhibition. It should be noted that aIN inhibition, which is more unreliable and smaller than cIN inhibition, remains largely unaffected by one-sided silencing, but it can potentially support dIN rebound firing. The failure of rhythmic activity suggests, however, that aIN inhibition is not sufficient to cause dIN rebound firing on its own.

The discrepancy between the one-sided fast silencing experiments and previous hemicord studies may be explained by the occurrence of homeostatic changes after hemisectioning, which facilitate pacemaker mechanisms to mediate motor rhythms. Previous hemicord studies normally leave at least a 20 min gap between the surgery and recording. We show here that, when the inhibition was blocked by strychnine and SR95531, the usual single-skin stimulation for evoking swimming failed to initiate any motor rhythm. Double-skin stimulation, which also evokes swimming in control conditions, led to seizure-like depolarization in most cases. The occasional rhythms, observed during the recovery period, suggest that the swimming circuit is still capable of generating rhythms, most likely via pacemaker firing in dINs. The delay in recordings after hemicord sections in previous studies is comparable to the recovery time revealed here (∼23 min), though the rhythms in the former were initiated by stimulating the hindbrain directly. It is not known whether the changes following hemisectioning and disinhibition by strychnine are similar or not. Whatever occurs during the recovery period to allow the resumption of rhythms, based on dIN pacemaker properties, is also unknown. As we show, individual dINs are capable of pacemaker firing throughout (Figures 6A and 6B). However, there is increased excitation in the absence of inhibition resulting from skin stimulation during the recovery period. Hyperexcitation in the presence of strychnine could have prevented dIN pacemaker firing during the recovery period (Figure 7). The homeostatic changes after disinhibition are therefore not readily understood in terms of a simple upregulation of pacemaker properties. There may be a change in fast homeostatic scaling of excitatory synaptic transmission (see, e.g., Frank et al., 2006) to overcome depolarization block of dIN pacemaker firing. Alternatively, some outward currents could be upregulated to allow pacemaker firing at higher excitation levels.

The two methods used in this study, optogenetics and −DC injections, enabled us to depress neuronal activity, including cIN inhibition, on a millisecond time scale, potentially leaving little time for reliable dIN pacemaker firing to become established in a substantial number of dINs to support the normal swimming rhythm. It is hard to exclude the presence of pacemaker firing during normal swimming, because dINs can, very occasionally, fire extra action potentials after the activity on the targeted side has stopped due to one-sided silencing. But such firing is very rare (Figures 3C and 3D). There is no difference in the tonic inward current, which gives rise to background excitation, between the failed cycle and the preceding cycle (Figures 5B and 5D). This means that, during normal swimming evoked by skin stimulation, the excitation level is not sufficient to drive pacemaker firing in most dINs. This is different from the failure of rhythm during pharmacological blockade of inhibition, in which double-skin stimulation evoked hyperexcitation and consequently blocked dIN pacemaker firing. The excitation levels in motor rhythms induced by (1) NMDA (and/or 5-HT) or (2) direct stimulation of hindbrain, reticulospinal formation, and spinal cord in many studies, including our own (Li et al., 2010), were artificially set by chemical concentrations or stimulation intensities. The millisecond silencing methods used in this study therefore provide experimental manipulations that conventional methods are incapable of achieving and can help reveal neural mechanisms occurring in normal conditions.

### The Role of Reciprocal Inhibition in Locomotion in Other Vertebrates

In another, more established swimming vertebrate model, zebrafish larvae, reciprocal inhibitory interneurons have been recently categorized (Higashijima et al., 2004; Liao and Fetcho, 2008). A subtype of commissural interneurons directly activated by the Mauthner cell has been shown to be only involved in escape response (Satou et al., 2009). The role of commissural interneurons that are rhythmically active during zebrafish swimming, however, has not been investigated.

Rapid progress has been made recently in unravelling the mammalian locomotor circuits (Goulding, 2009; Grillner and Jessell, 2009; Kiehn, 2006). Although the core composition of locomotor rhythm generation circuit has not been clearly defined, it is well known that there is reciprocal inhibition between flexor and extensor pathways and between the left and right side of the spinal cord (Kiehn, 2006). Some of the reciprocal inhibition between the left and right sides is polysynaptic, involving cross-excitatory interneurons (Kjaerulff and Kiehn, 1997; Butt and Kiehn, 2003). Blocking glycinergic inhibition transforms alternating flexor-extensor and left-right activity into bilateral synchronous motor rhythms (Beato and Nistri, 1999; Cowley and Schmidt, 1995). This has led to the suggestion that excitatory networks are central to mammalian locomotor rhythm generation and that half-center modules are deemed obsolete (Stein and Smith, 1997; Kiehn, 2006). Reciprocal inhibition is still believed to play some role in mammalian locomotor rhythm generation, because the burst intervals in strychnine are longer than those seen in intact preparations (Kjaerulff and Kiehn, 1997).

### Conclusions

Our study provides strong evidence that reciprocal inhibition is not only important in coordinating activity between the left and right sides of the spinal cord, but is also essential in the maintenance of normal swimming rhythm. This view is based on silencing neuronal activity very rapidly, at a speed that cannot be achieved with other approaches, such as lesioning, pharmacological blockade, and genetic ablations.

## Experimental Procedures

*Xenopus* embryos were collected and raised after regular human chorionic gonadotropin injections to pairs of adult *Xenopus*. All experimental procedures were approved by a local Animal Welfare Ethics committee and comply with UK Home Office regulations. Tadpoles at 2 days old (stage 37/38) were immobilized using α-bungarotoxin (12.5 μM, Tocris). Recording saline contained 115 mM NaCl, 3 mM KCl, 2 mM CaCl_2_, 2.4 mM NaHCO_3_, 1 mM MgCl_2_, 10 mM HEPES, with pH adjusted to 7.4 with NaOH. Dissections were made to expose neuronal cell bodies in the tadpole spinal cord and hindbrain for whole-cell recordings. Tadpole hindbrain was cross-sectioned at the fifth and sixth rhombomere levels to remove all higher brain inputs to the swimming circuit in most experiments. Electrode solution (concentrations: 100 mM K-gluconate, 2 mM MgCl_2_, 10 mM EGTA, 10 mM HEPES, 3 mM Na_2_ATP, 0.5 mM Na guanosine triphosphate adjusted to pH 7.3 with KOH) contained 0.1% neurobiotin (Vector Labs) for final identification of neurons after recordings. Whole-cell recordings were made in either current-clamp or voltage-clamp mode with an Axon Multiclamp 700B, digitized with a Power 1401 mkII, and sampled with Signal (version 4, CED). We applied electrical stimulus (0.2 ms) to the tail skin of immobilized tadpoles to start fictive swimming (defined as swimming throughout the text). m.n. recordings were made with glass suction electrodes from the middle trunk region on the left. A swimming cycle is the period from one m.n. burst to the next one. For injecting −DC into single dINs via the recording electrode, the −DC (rectangular pulses) level was progressively increased without membrane destabilization. In the dINs in which −DC could stop swimming, the absolute amplitude of DC was about three to six times the threshold current for evoking dIN firing at rest. The dIN membrane potential was hyperpolarized by a maximum of 150 mV.

To evoke rebound firing in dINs at rest, we placed a suction electrode on one side of the spinal cord surface to stimulate cINs directly and dINs were recorded on the opposite side. Saline containing a combination of 6 μM NBQX (AMPA receptors, 2, 3-dihydroxy-6-nitro-7-sulfamoylbenzo-[f]quinoxaline), 60 μM D-AP5 (D-(-)-2-amino-5-phosphonopentanoic acid, NMDAR, Tocris), and 2 μM DHβe (nicotinic receptors, Dihydro-β-erythroidine, Research Biochemicals International) was microperfused close to the recorded dIN to block excitatory synaptic transmission from sensory pathway neurons. To test dIN pacemaker firing in intact tadpoles, we applied NMDA (100 mM, prepared with equimolar sodium hydroxide) within 50 μm upstream to the recorded neuron using microelectrode iontophoresis (Li et al., 2010). TTX (0.4 μM) was bath applied to block action potentials, while 100 μM NMDA was applied by microperfusion to evoke TTX-resistant oscillation in dINs (Li et al., 2010). Strychnine and SR95531 (gabazine, Tocris) were bath applied.

ArCh complementary DNA (Chow et al., 2010) was obtained from Addgene. The open reading frame was tagged with GFP and inserted into a *Xenopus* expression vector, incorporating a T7 promoter, a restriction site for linearization, and both 3′ and 5′ *Xenopus* globin-flanking sequences that aid translation and stabilize the message. cRNA was transcribed in vitro using Ambion mMessage mMachine. cRNA concentration was measured using a NanoDrop ND-1000 spectrophotometer. ArCh cRNA (0.5–1 ng) was injected into one blastomere of two- to eight-cell stage embryos showing regular cleavage patterns, using a TooheySpritzer (Toohey Company). The embryos then were raised to stage 37/38 and their GFP expression examined. Tadpoles with good GFP expression on the right side were chosen for light-silencing tests and electrophysiological recordings. Yellow light for ArCh activation and blue light for GFP observation from LED arrays (pE-1, CoolLED) were controlled by Power 1401 mkII using Signal software. A Nikon E600 FN or an Olympus BX51WI microscope was used for visually guided patch-clamp recordings. Yellow light (wide field, typically 1 s in duration) was applied through a 40× water-immersion objective with a maximum intensity of 10 mW/mm^2^ onto the caudal hindbrain area of tadpoles. The intensity of light was gradually increased until we found a level that could reliably stop swimming. Fluorescent images were captured using a Veho VMS-004 USB microscope or a Scientifica BFWCAMXM camera mounted on the recording microscope.

Data processing and analyses were carried out using Dataview (v6.1, courtesy of Dr. W.J. Heitler in the University of St. Andrews) and Excel. Statistical analyses were carried out using IBM PASW statistics 18 (SPSS). For normally distributed data, mean was given with standard error and statistical differences between groups were examined using Student’s t test. For nonnormally distributed data, median and range were given and measurements were compared using the Wilcoxon signed-rank test. The effectiveness of one-sided silencing (either yellow light illumination or −DC injections into dINs) was assessed in trials alternated with control episodes at the beginning of each recording. A side was judged active if regular m.n. bursts were recorded or neurons received rhythmic excitatory synaptic potentials/currents.
