# Supplementary material for: Fast Silencing Reveals a Lost Role for Reciprocal Inhibition in Locomotion
Source: Neuron. 2013 Jan 9;77(1):129–40. doi: 10.1016/j.neuron.2012.10.040 (PMC3542422; doi:10.1016/j.neuron.2012.10.040)
Supplement: Document S1. Figure S1 [file mmc1.pdf]

Neuron, Volume *77*

Supplemental Information

Fast Silencing Reveals a Lost Role for Reciprocal Inhibition in Locomotion

Peter R. Moult, Glen Cottrell, and Wen-Chang Li

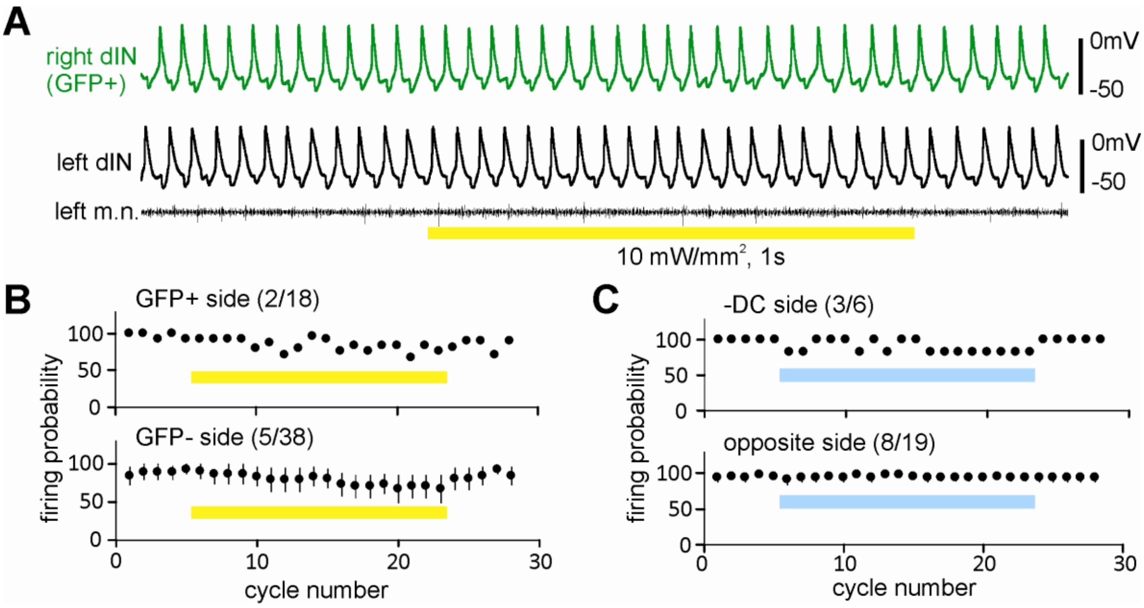

Figure S1. Cycle-by-cycle neuronal firing probability did not drop significantly in trials where one-sided silencing failed to stop swimming. (A) An example trial when light failed to decrease neuronal firing probability (right and left dIN) and stop swimming (left m.n.). Note the reliable mid-cycle inhibition in both dINs. (B**)** Neuronal firing probability in light illumination experiments. Yellow bars indicate 1 second period of illumination. (C) Firing probability in –DC injection experiments. Blue bars indicate 1 second of current injections. Numerals in brackets are number of cells/trials, respectively. Vertical bars are standard errors. Cycle-by-cycle firing probability was first calculated for each cell and then averaged. One second period of one-sided silencing lasted for 18-21 cycles and only the first 17 cycles were used for averaging.

The continuous neuronal firing/swimming during silencing period means that reciprocal inhibition was not significantly suppressed as expected in these cases. Comparing probability in the last 5 cycles during silencing with control revealed no significance in all cases (GFP+ side and –DC side data pooled together, $p>0.05$, Wilcoxon Signed Rank test in each case).
